# Promising results of captopril in improving knee arthrofibrosis and cartilage status: an animal model study

**DOI:** 10.1186/s40634-022-00516-5

**Published:** 2022-07-28

**Authors:** Seyed Ali Hashemi, Ali Azad, Amirhossein Erfani, Reza Shahriarirad, Negar Azarpira

**Affiliations:** 1 Research Center for Bone and Joint Diseases, Department of Orthopedic Surgery, Chamran Hospital, Shiraz University of Medical Sciences, Shiraz, Iran; 2 Student Research Committee, Shiraz University of Medical Sciences, Shiraz, Iran; 3 Thoracic and Vascular Surgery Research Center, Shiraz University of Medical Sciences, Shiraz, Iran; 4 Transplant Research Center, Shiraz University of Medical Sciences, Shiraz, Iran; 5 Nanomedicine and Nanobiology Research Center, Shiraz University of Medical Sciences, Shiraz, Iran

**Keywords:** Captopril, Arthrofibrosis, Animal modeling, Osteoarthritis

## Abstract

**Purpose:**

Several cytokines and growth factors start and progress the destruction process of joint hyaline cartilage and fibrosis formation. Captopril is classified as an Angiotensin-converting enzyme inhibitor in which several studies revealed that captopril significantly decreases fibrosis formation in some organs like the liver, heart, and kidney. This study aimed to evaluate the use of captopril in reducing the possibility of arthrofibrosis and osteoarthritis in an animal model.

**Method:**

In this in-vivo animal model study, the anterior cruciate ligament of 24 rabbits was transected to induce osteoarthritis and arthrofibrosis. The control group contained 11 rabbits and the second group consisted of 13 rabbits. The second group was treated with 10 mg/ kilogram/day captopril through a nasogastric tube. The control group was treated with normal saline in the same way. Cartilage damage and osteoarthritis were evaluated by Osteoarthritis Research Society International (OARSI) scoring system. After 30 days, animals were sacrificed, and arthrofibrosis and cartilage damage were evaluated microscopically and macroscopically.

**Results:**

According to macroscopic and microscopic evaluation, captopril dramatically reduced arthrofibrosis formation based on visual scoring and the Masson trichrome staining system. Cartilage damage was lower in the intervention group compared to the control group.

**Conclusions:**

Captopril is an angiotensin-converting enzyme inhibitor that demonstrated to significantly decreases the possibility of arthrofibrosis. Although the beneficial preventive effect of captopril on osteoarthritis was not proved statistically, better results may be obtained if the route of administration or drug dosage is changed.

## Background

Arthrofibrosis is one of the main problems of post-joint trauma or surgery that causes a decrease in a patient’s function due to loss of joint mobility [13]. It is defined as scarring, fibrotic response, and cellular hyperplasia in the joint space [22, 30]. The most effective treatment of arthrofibrosis is surgical release. Some authors suggested pharmacological treatments to prevent scar and fibrous tissue formation, such as corticosteroid administration, intraarticular chitosan injection, local administration of Fibroblast growth factors (FGF) antibodies, and a hyaluronan derivative gel. The results of such treatments are not yet evident in clinical trials [2, 8, 19].

Osteoarthritis (OA) is a joint degenerative process that decreases patients’ daily functions by the mechanism of a gradual loss of joint cartilage matrix [25]. Several cytokines and growth factors progress the destruction process of the joints’ hyaline cartilage. Angiogenesis and inflammation play an important role in the beginning and facilitating the joint degenerative process through various mediators [25]. The most important cytokines are interleukin 1 (IL1) and tumor necrosis factor (TNF), produced by the chondrocyte and synovium. Matrix metalloproteinase (MMP) mediates and facilitates the destructive effect of IL1 and TNF on the joint surface. This destructive process can be stopped by the blockage of MMP function in several routes [25].

Angiotensin II is produced and secreted by endothelial cells by the converting effect of the angiotensin-converting enzyme (ACE) and facilitates fibrosis formation by induction of transforming growth factor (TGF), which is also a fibrous forming factor. Angiotensin-converting enzyme inhibitor (ACEI) decreases the production of angiotensin II, subsequently decreasing the production of IL1, TNF, MMP, TGF, and vascular endothelial growth factor (VEGF). Several studies revealed that ACEI significantly reduces fibrosis formation in some organs, including the liver, heart, and kidneys [3, 28, 31, 33, 38]. Studies suggest that this drug family may affect the renin angiotensin system by inhibiting angiotensin II, stimulating prostaglandin and bradykinin, and showing antioxidant effect through the free radical scavenger action [5].

Captopril, classified as an ACEI, has been proven to act as a potent anti-fibrotic and anti-inflammatory drug in several studies by decreasing the production and induction of TGF and FGF [3, 10, 16, 17, 31, 33, 40]. Studies have demonstrated that captopril is effective in preventing colonic fibrosis in TNBS-induced colitis due to the blockade of TGFβ-1 overexpression and a direct down-regulation of TGFβ-1 transcript [35]. Also, captopril has been showed to be effective against liver fibrosis [17].

This study aimed to evaluate the use of captopril in decreasing the possibility of arthrofibrosis and OA in an animal model. The study’s proposed animal model was rabbits, which is widely validated in investigating OA disease since it determines pathological and biomechanical changes similar to those seen in humans [34, 39].

## Materials and methods

### Study design

This study was an in-vivo animal model study. All experimental protocols were approved by the Institutional Animal Care and the Ethics Committee of Shiraz University of Medical Sciences, Shiraz, Iran (Ethical code: IR.SUMS.MED.REC.1391.4014). All procedures conformed to the guidelines for the care and handling of animals prepared by the Iranian ministry of health and medical education and in accordance with the international conventions on animal experimentation.

### Animals

Twenty-four male Dutch rabbits weighing between 1.7 and 2.3 kg were obtained. Rabbits were placed in separated cages at room temperature ranging from 25 ± 2° c with a dark and light cycle of 12:12 hours. Animals had free access to food and water throughout the experiment. The rabbits were caged together in specific and separated groups. The cages were large enough in order not to limit the activity of rabbits.

### Surgery

To induce OA and arthrofibrosis according to standard models, all animals were anesthetized by intramuscular injection of 2% xylazine hydrochloride in a dose of 8 mg/kilogram (mg/kg) and 5% ketamine hydrochloride in a dose of 10 mg/kg. Before incision, 50 mg/kg of cefazolin sodium was injected intramuscularly to prevent infection. The operation site was shaved. Surgery was performed through an anterior midline longitudinal incision on the right knee. After opening the fascia, the joint was opened through the medial parapatellar approach, the patella and the patellar tendon were reflected laterally, and the anterior cruciate ligament was explored and cut with a scalpel number 11. A partial synovectomy was done. After joint irrigation with normal saline, the medial patellar retinaculum and the skin were closed by 3–0 vicryl and 3–0 nylon sutures, respectively [15, 19, 21, 24, 42].

### Grouping and intervention

After applying the sterile dressing, rabbits were categorized into two main groups; the first group consisted of 11 rabbits as a control group that was being treated with normal saline through a nasogastric tube, and the second group consisted of 13 rabbits that were treated with 10 mg/kg/day captopril [12]. Captopril with a dose of 10 mg/kg/d was dissolved in 20 ccs of drinking water and was transfused into the nasogastric tube every morning at 8 o’clock. Since arthrofibrosis formation takes 4 weeks after transection of the anterior cruciate ligament and partial synovectomy, treatment continued for 30 days with no sign of septic arthritis, cellulitis, or any other wound problem during the study.

Then rabbits were sacrificed with high doses of Pentothal (200 mg/kg) 30 days after treatment.

After euthanization, joints were opened through the previous incision and observed by the same surgeon who operated on the rabbits, in a double-blinded fashion.

### Macroscopic evaluation

A visual scoring system ranged from zero to three [19], including Score Zero: No Adhesions, Score One: Weak, mild, filmy adhesions that can be eliminated by minimal manual traction, Score Two: Moderate adhesion that can be eliminated by manual traction, Score Three: Dense fibrous adhesions that must be surgically removed. This scoring method was initially described by Rothkopf et al. [29] and has been used in various animal studies [4, 9, 11, 18, 24, 41].

### Microscopic evaluation

Tibias and femurs were fixed in 10% formalin for 7 days, then transferred to a decalcifier (Nitric acid 5%) for 2 days. The tibias and femurs were disarticulated, trimmed in the frontal plane, and embedded in paraffin for sectioning at 8 μm. Sections were stained with hematoxylin and eosin, safranin red-O, and Masson’s trichrome (for evaluation of fibrosis). Stained sections were evaluated microscopically by a specialized pathologist blinded from the groups and cases. Detection of the fibrous adhesion band was considered positive if the pathologist could microscopically detect the Masson trichrome blue fibers on the joint surface [19].

The Osteoarthritis Research Society International (OARSI) scoring system was utilized in this study [27]. This scoring system is a pathology assessment system based on six grades, which reflect the depth of the lesion and four stages reflecting the extent of OA over the joint surface. The tibial and femoral bone, as well as frontal tissue for evaluating arthrofibrosis and OA. In this study, the medial compartment containing the medial tibial and femoral condyles was evaluated.

**Fig. 1:**
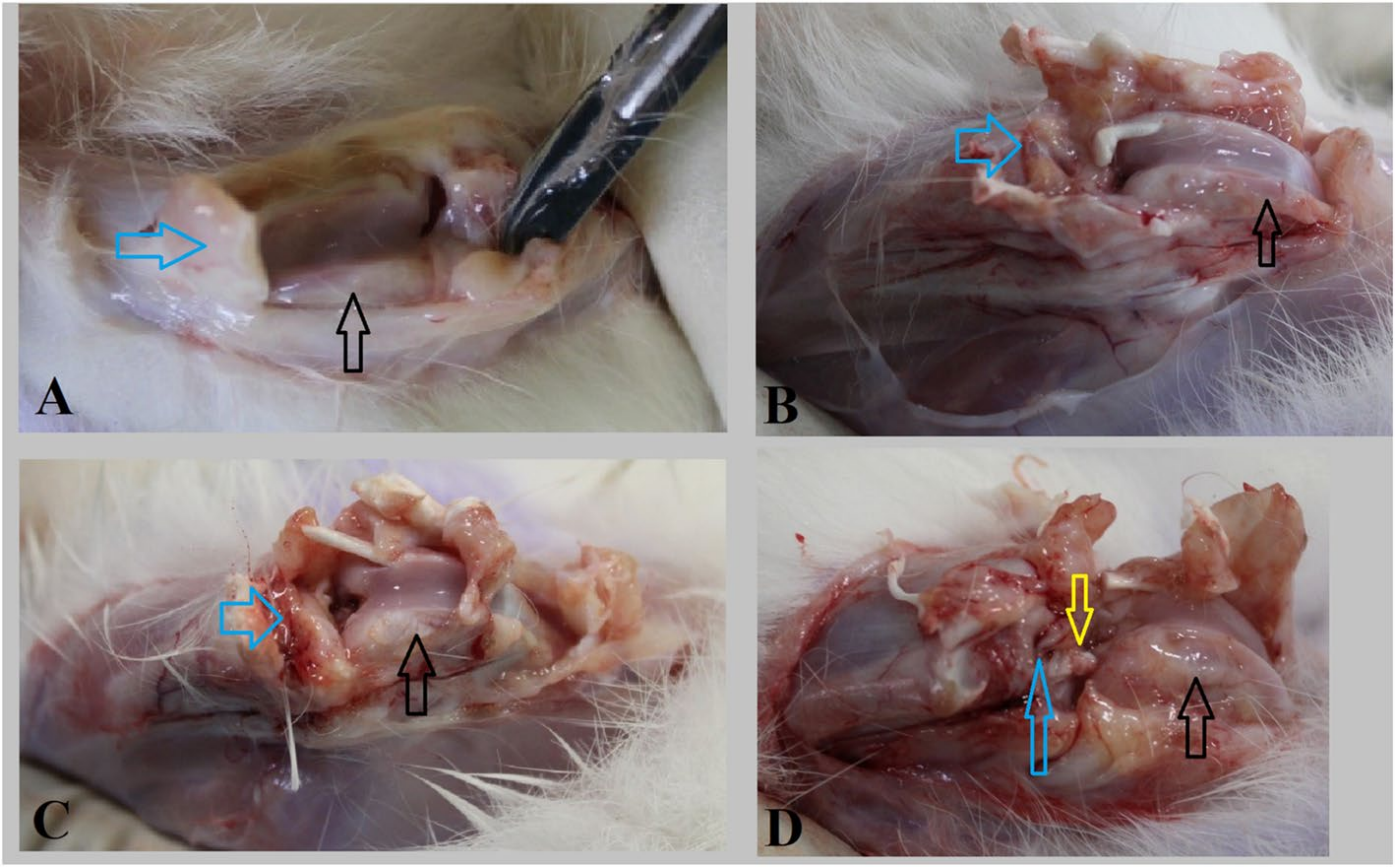
**A** Stage Zero of adhesion band formation with visual score system assessment, note that there is no adhesion band seen, Also no synovium proliferation is observed. **B** Stage one of adhesion band formation with visual score system assessment, note that there is minimal adhesion band formed. **C** Stage two of adhesion band formation with visual score system assessment, note that there is moderate adhesion band formation needs manual traction for releasing. **D** Stage three of adhesion band formation with visual score system assessment, note that joint is completely stiff and needs surgical release, while also synovium proliferation is observed; (Black arrow indicator of the femoral articular surface; Blue arrow indicator of the tibial articular surface; Yellow arrow indicator of dense adhesion band)

The section was extended horizontally from one edge of the joint to the other and in-depth from the articular surface to below the articular bone plate. The grade is defined as the depth of cartilage damage. The grade is indicative of the severity of the destruction process. The stage is defined as the horizontal extent of cartilage involvement within one side of a joint compartment. To examine microscopic changes, the OARSI cartilage histopathology assessment system was used, in which grading ranged from zero to six (Table 1) and staging from zero to four (Fig. 1) [27]. Stage one represents less than 10% involvement. Stage two represents 10–25% involvement. Stage three represents 25–50% involvement. Stage four represents more than 50% involvement (Fig. 1).

**Table 1:** Key feature of different grades based on the Osteoarthritis Research Society International scoring system

Grade	Key feature	Associated criteria (tissue reaction)
**Zero**	Surface intact, cartilage morphology intact	Matrix: normal architecture Cells: intact, appropriate orientation
**One**	Surface intact	Matrix: superficial zone intact, edema and/or superficial fibrillation (abrasion) Cells: death, proliferation (clusters), hypertrophy
**Two**	Surface discontinuity	Matrix: discontinuity at superficial zone Cells: death, proliferation (clusters), hypertrophy
**Three**	Vertical fissures (clefts)	Matrix: vertical fissures into the mid zone Cells: death, regeneration (clusters), hypertrophy
**Four**	Erosion	Matrix: matrix loss of superficial layer and mid zone
**Five**	Denudation	Surface: sclerotic bone or reparative tissue including fibrocartilage within the denuded surface. Microfracture with repair limited to the bone surface
**Six**	Deformation	Bone remodeling more than osteophyte formation only

### Statistical analysis

Collected data were analyzed by SPSS Ver. 22 (SPSS Inc., Chicago, IL, USA). After applying chi-square and Fisher’s exact test was used to evaluate the significant association among categorical variables. A *P*-value of under 0.05 was considered significant.

## Results

### Surgical observations

In this experimental model of OA and fibrosis, marked synovial thickening as a result of diffuse fibrous tissue proliferation in both medial, lateral, and patellar synovium is obvious. There are large cartilage lesions on the anterior femur where the tibia is sliding forward, and finally, osteophytes, especially around the patellar groove is developed. The treatment with captopril has resulted in visually less thickening of the overall synovium, as demonstrated in Fig. 1. Also, in gross evaluation, after dissection of the joints, there was macroscopic evidence of joint and cartilage fibrosis in the case group. These joints were stiffer and required more manual traction to gain access to the joint surfaces, and adhesion bands covering cartilage were primarily noticed in this group.

### Macroscopic evaluation

Macroscopic evaluation of the fibrosis formation after applying the visual scoring system is shown in Table 2. According to the visual scoring system, more than half of the rabbits (9 out of 11) in the control group had stage two and three fibrosis macroscopically compared to the group treated by captopril, and less than half of the rabbits having higher stages. A significant difference was found statistically in the macroscopic evaluation of adhesion band formation (*P*-value< 0.05).

**Table 2:** Comparison of macroscopic and microscopic findings of subjects treated with captopril (case group) and subjects treated with normal saline (control group) based on arthrofibrosis

Variables	Case group; *n = 13*	Control group; *n = 11*	***P***-value*
**Macroscopic**			
*Score Zero*	1 (7.7%)	0 (0%)	0.003
*Score One*	6 (46.2%)	2 (18.2%)	
*Score Two*	6 (46.2%)	2 (18.2%)	
*Score Three*	0 (0%)	7 (63.6%)	
**Microscopic**			
*Positive*	5 (38.5%)	10 (90.9%)	0.013
*Negative*	8 (61.5%)	1 (9.1%)	

*Chi-square/Fisher’s Exact test

### Microscopic evaluation

As demonstrated in Table 2, all rabbits in the control group were Masson’s trichrome stain positive except one rabbit. In the case group, eight rabbits without arthrofibrosis were included. All of the results’ differences were statistically significant (*P*-value = 0.013).

**Fig. 2:**
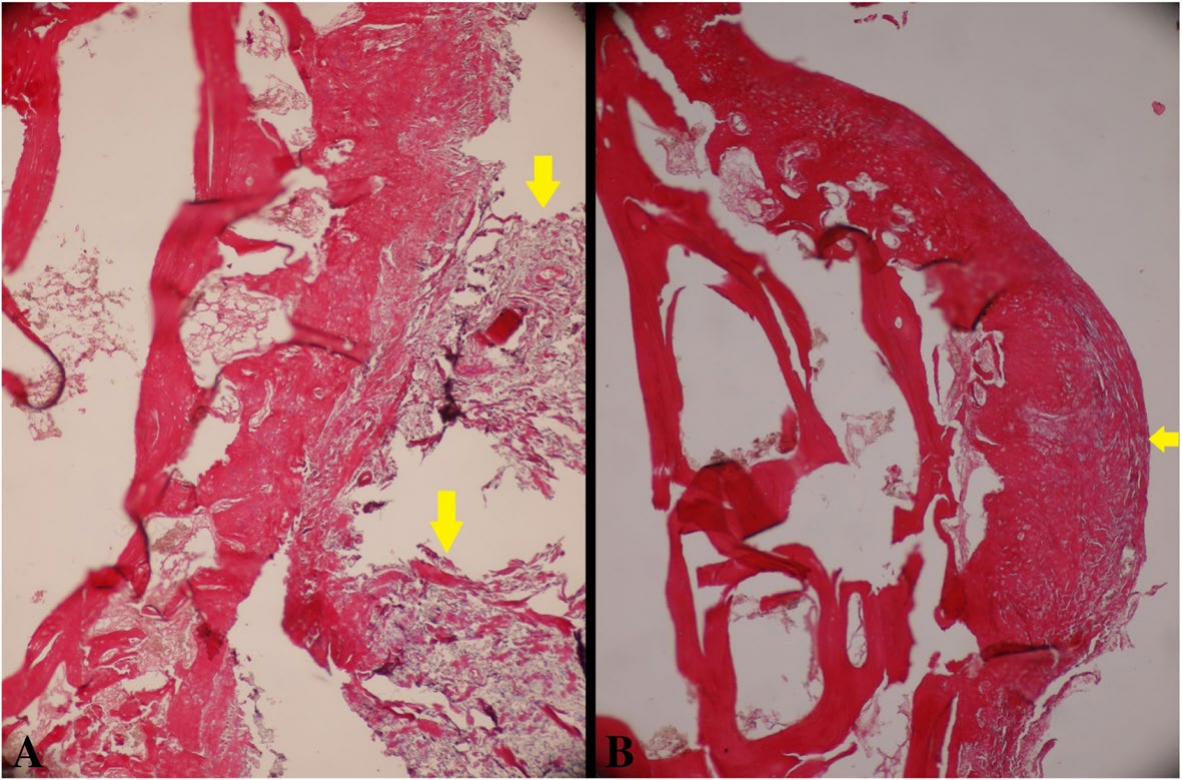
**A** Control group with marked arthrofibrosis and deposition of collagen bundles in articular surface (yellow arrow); **B** Treatment group with captopril, the collagen deposition is less obvious (× 40)

**Fig. 3:**
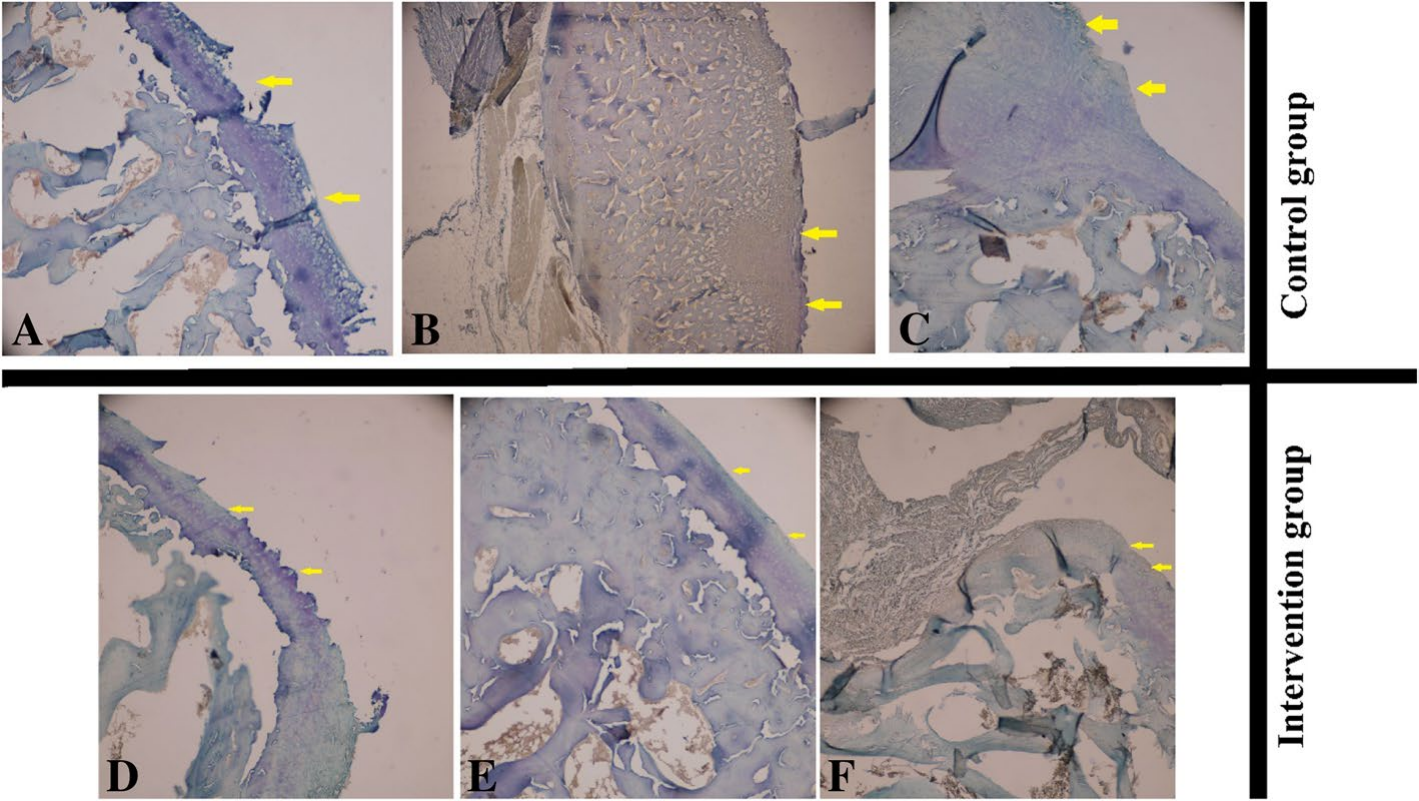
Control group: **A** Grade Four; **B** Grade Five; **C** Grade Six; Intervention group: **D** Grade Three; **E** Grade Four; **F** Grade Five. Articular surface damage is obvious in both the control and intervention groups; however, the severity of damage is more severe in the control group. After the intervention, the severity of fibrosis is decreased with less articular damage (×40) (yellow arrow indicator of articular surface)

Eleven rabbits in the second group were categorized in grade four of destruction, and there were only two rabbits with high grades of destruction (grade five), but in the control group, there were four rabbits with high grades of destruction (grade five). According to the results, the staging and grading scores of OA were better in the case group, however, there was no significant difference statistically, except in the grading of femoral sections which worsened in the case group (*P*-value = 0.001). There was no statistical difference among the two groups regarding the staging of femoral sections, grading, and staging of tibial sections (*P*-value = 0.066, 0.058, and 0.458, respectively). Microscopic evaluation of the fibrosis formation and OA after applying the visual scoring system is shown in Tables 2 and 3. Also, Fig. 2 shows negative and positive results for arthrofibrosis. Also, Fig. 3 shows different grades of OA microscopically in the intervention and control groups.

**Table 3:** Comparison of microscopic findings of subjects treated with captopril (case group) and subjects treated with normal saline (control group) based on osteoarthritis

Variables	Case group; *n = 13*	Control group; *n = 11*	***P***-value*
**Femoral**			
Grade			
*Three*	0 (0%)	5 (45.5%)	0.001
*Four*	11 (84.6%)	2 (18.2%)	
*Five*	2 (15.4%)	4 (36.4%)	
Stage			
*Two*	4 (30.8%)	0 (0%)	0.066
*Three*	9 (69.2%)	9 (81.8%)	
*Four*	0 (0%)	2 (18.2%)	
**Tibial**			
Grade			
*Three*	1 (7.7%)	1 (9.1%)	0.058
*Four*	10 (76.9%)	3 (27.3%)	
*Five*	2 (15.4%)	6 (54.5%)	
*Six*	0 (0%)	1 (9.1%)	
Stage			
*Two*	4 (30.8%)	0 (0%)	0.066
*Three*	9 (69.2%)	9 (81.8%)	
*Four*	0 (0%)	2 (18.2%)	

*Chi-square/Fisher exact test

## Discussion

The most important findings of the present study were that captopril as an angiotensin-converting enzyme inhibitor attenuates arthrofibrosis by interfering in the fibrosis formation process and decreasing the production of some important cytokines. Although the method of evaluation of the arthrofibrosis that was applied was one of the standard methods, further studies must be done to evaluate the fibrosis formation in detail according to the cellular aspect like fibroblast proliferation and collagen synthesis with other ACEIs and different dosage or route of administrations. Furthermore, although the beneficial preventive effect of captopril on OA was not proved statistically, it seems that it may decrease the possibility of cartilage damage if the drug is applied through another route of administration or with a higher dosage.

Captopril demonstrated macroscopical and microscopically improved the attenuation of fibrosis formation in joints. This mechanism is mainly through decreasing the production of IL1, TNF, MMP, TGF, FGF, and VEGF [7, 18, 26, 36]. Several studies revealed the potent effect of captopril as an anti-fibrotic agent. Jonson et al. reported that captopril significantly attenuates the progression of hepatic fibrosis and decreases fibrosis formation of the liver in rats, and there is a strong relationship between the level of TGF, angiogenesis, and progression of fibrosis formation [16]. Another study by Karimian et al. also confirmed that enalapril decreases the progression of fibrosis in the liver with its significant antioxidant effect while also reporting 10 mg/kg/day of captopril as the minimum effective dose [17]. Although our results demonstrated captopril’s potential for decreasing OA and arthrofibrosis, further studies regarding its molecular pathway are required to provide a better understanding and administration properties of this medication.

Captopril interferes with some degenerative processes and prevents them. A study by Yang et al. demonstrated that captopril exerted chondroprotection in a rat model of OA and inhibited cartilaginous degeneration [32]. Tang et al. [32] observations of elevated RAS component expression and suppressed Angiotensin II expression in the proximal tibia of OA rats. They also showed that taking captopril reduced osteoarthritic lesions by boosting Angiotensin II transcription. Similarly, Aliskiren (a renin inhibitor)-modified local RAS of the knee in OA was similarly followed by a decrease in renin, ACE, Angiotensin II and its type I receptor expression and an increase in angiotensin type II receptor [37]. These in vivo and in vitro studies suggest that enhanced expression of the Angiotensin II receptor may be involved in the chondroprotection linked to RAS inhibition. This supports the idea that this receptor is involved in the protection brought on by ACE inhibition in OA [6]. A recent study also supported the role of local RAS in all knee joint tissues, including cartilage, synovium, and bone, along with Angiotensin II harmful effects on chondrocytes and joint tissues. The idea that Ang II plays a substantial role in the pathogenesis of OA is supported by the fact that intra-articular injections of the substance cause joint edema, joint hyperalgesia, and an increase in the number of leukocytes in the joint cavity. These findings confirmed the favorable effects of ACE inhibition with captopril in chondrocyte culture in an OA model, showing that local RAS regulation can influence the development of OA [6]. As predicted, in our study, the degenerative process of the captopril group was milder than the control group, except for the femoral sections’ OA, however not significant. Based on the staging, captopril had a beneficial effect on the extent of femoral condyle destruction. There were four rabbits with stage two and no rabbits with stage four in the second group, while in the control group, two rabbits with a higher stage of destruction were observed. Therefore, captopril decreases the degenerative process in the femoral condyle, although no statistical significance was achieved; However, the clinical importance cannot be underestimated and requires further human and more extensive populational studies. The medial tibial condyle microscopically was assessed while applying a grading and staging system similar to the femoral condyle. The overall results of the captopril group were better than the control group, however, without achieving statistical significance. Eleven rabbits with lower grades three and four were in the second group compared with only four in the control group. Similar results are concluded while evaluating the depth of destruction in the medial tibial condyle.

The most suitable method for diagnosing and treating joint disorders is still a matter of debate [1, 8, 14]. Several studies supported permitting the administration of captopril in normotensive patients without significant complications [20, 23]. In our study, captopril was administered through a nasogastric tube. This may cause a decrease in the availability of the drug in the rabbit’s circulation system. It is predictable that administration of the medication intravenously or intra articular would provide more improved results.

### Limitations

Among the limitations is the evaluation of the possibility of arthrofibrosis and OA during an early postoperative period which might not correlate with the disease degree that perseveres after longer intervals. However, the point of evaluating the possibility of arthrofibrosis and OA in an early postoperative term was related to animal care and the limitations of the used model. Hence further studies are demanded to investigate the influence of captopril on arthrofibrosis and OA after longer intervals associated with the clinical practice. Also, the systemic side effects of the studied drugs should be evaluated, and their arthrofibrosis and OA prevention properties should be weighed against their consequences. Furthermore, further studies in human subjects and larger populations are needed to confirm captopril’s efficacy in treating arthrofibrosis and OA. Finally, our study did not assess the cellular immune pathway and related cytokines and chemokines and their direct role in arthrofibrosis and OA formation, which warrants further molecular and immunocytological studies.

## Conclusion

Captopril showed promising results in reducing arthrofibrosis and cartilage damage. Further larger sample studies and human studies are warranted to evaluate the fibrosis formation in detail according to the cellular aspect, along with alternative dosages and application methods.

## Data Availability

All data regarding this study has been reported in the manuscript. Please contact the corresponding author if you are interested in any further information.

## References

[1] Bhimani R, Shahriarirad R, Ranjbar K, Erfani A, Ashkani-Esfahani S (2021). Transportal versus all-inside techniques of anterior cruciate ligament reconstruction: a systematic review. J Orthop Surg Res.

[2] Bosch U (2002). Arthrofibrosis. Orthopade.

[3] Brower GL, Levick SP, Janicki JS (2007). Inhibition of matrix metalloproteinase activity by ACE inhibitors prevents left ventricular remodeling in a rat model of heart failure. Am J Physiol Heart Circ Physiol.

[4] Brunelli G, Longinotti C, Bertazzo C, Pavesio A, Pressato D (2005). Adhesion reduction after knee surgery in a rabbit model by Hyaloglide®, a hyaluronan derivative gel. J Orthop Res.

[5] Costa LE, La-Padula P, Lores-Arnaiz S, D’Amico G, Boveris A, Kurnjek ML (2002). Long-term angiotensin II inhibition increases mitochondrial nitric oxide synthase and not antioxidant enzyme activities in rat heart. J Hypertens.

[6] de Sa GA, Dos Santos A, Nogueira JM, Dos Santos DM, Amaral FA, Jorge EC (2021). Angiotensin II triggers knee joint lesions in experimental osteoarthritis. Bone.

[7] Di Nicola V (2020). Degenerative osteoarthritis a reversible chronic disease. Regen Ther.

[8] Ebrahimi AAH, Pourfraidon Ghasrodashti Z, Tanide N, Shahriarirad R, Erfani A, Ranjbar K, Ashkani-Esfahani S (2021). Therapeutic effects of stem cells in different body systems, a novel method that is yet to gain trust: a comprehensive review. Bosn J Basic Med Sci.

[9] Emami MJ, Jaberi FM, Azarpira N, Vosoughi AR, Tanideh N (2012). Prevention of arthrofibrosis by monoclonal antibody against vascular endothelial growth factor: a novel use of bevacizumab in rabbits. Orthop Traumatol Surg Res.

[10] Freeman TA, Parvizi J, Dela Valle CJ, Steinbeck MJ (2010). Mast cells and hypoxia drive tissue metaplasia and heterotopic ossification in idiopathic arthrofibrosis after total knee arthroplasty. Fibrogenesis Tissue Repair.

[11] Fukui N, Tashiro T, Hiraoka H, Oda H, Nakamura K (2000). Adhesion formation can be reduced by the suppression of transforming growth factor-β1 activity. J Orthop Res.

[12] Ghazi-Khansari M, Mohammadi-Karakani A, Sotoudeh M, Mokhtary P, Pour-Esmaeil E, Maghsoud S (2007). Antifibrotic effect of captopril and enalapril on paraquat-induced lung fibrosis in rats. J Appl Toxicol.

[13] Gollwitzer H, Burgkart R, Diehl P, Gradinger R, Bühren V (2006). Therapy of arthrofibrosis after total knee arthroplasty. Orthopade.

[14] Hashemi SA, Ranjbar MR, Tahami M, Shahriarirad R, Erfani A (2020). Comparison of accuracy in expert clinical examination versus magnetic resonance imaging and arthroscopic exam in diagnosis of meniscal tear. Adv Orthop.

[15] Hayami T, Pickarski M, Zhuo Y, Wesolowski GA, Rodan GA, Duong LT (2006). Characterization of articular cartilage and subchondral bone changes in the rat anterior cruciate ligament transection and meniscectomized models of osteoarthritis. Bone.

[16] Jonsson JR, Clouston AD, Ando Y, Kelemen LI, Horn MJ, Adamson MD (2001). Angiotensin-converting enzyme inhibition attenuates the progression of rat hepatic fibrosis. Gastroenterology.

[17] Karimian G, Mohammadi-Karakani A, Sotoudeh M, Ghazi-Khansari M, Ghobadi G, Shakiba B (2008). Attenuation of hepatic fibrosis through captopril and enalapril in the livers of bile duct ligated rats. Biomed Pharmacother.

[18] Kazemi K, Hosseinzadeh A, Shahriarirad R, Nikeghbalian S, Kamran H, Hosseinpour P (2022). Comparison of oral sirolimus, prednisolone, and combination of both in experimentally induced peritoneal adhesion. J Surg Res.

[19] Kocaoglu B, Akgun U, Nalbantoglu U, Poyanli O, Karahan M (2011). Adhesion reduction after knee surgery in a rat model by mitomycin C. Knee Surg Sports Traumatol Arthrosc.

[20] Mathiesen ER, Hommel E, Giese J, Parving HH (1991). Efficacy of captopril in postponing nephropathy in normotensive insulin dependent diabetic patients with microalbuminuria. BMJ.

[21] Matthews JL, Chung M, Matyas JR (2004). Indirect injury stimulates scar formation-adaptation or pathology?. Connect Tissue Res.

[22] Mayr HO, Fassbender FF, Prall WC, Haasters F, Bernstein A, Stoehr A (2019). Immunohistochemical examination in arthrofibrosis of the knee joint. Arch Orthop Trauma Surg.

[23] Mimran A, Insua A, Ribstein J, Bringer J, Monnier L (1988). Comparative effect of captopril and nifedipine in normotensive patients with incipient diabetic nephropathy. Diabetes Care.

[24] Namazi H, Torabi S (2007). Novel use of botulinum toxin to ameliorate arthrofibrosis: an experimental study in rabbits. Toxicol Pathol.

[25] Oliviero F, Ramonda R, Punzi L (2010). New horizons in osteoarthritis. Swiss Med Wkly.

[26] Paish HL, Kalson NS, Smith GR, del Carpio Pons A, Baldock TE, Smith N (2018). Fibroblasts promote inflammation and pain via IL-1α induction of the monocyte chemoattractant chemokine (CC motif) ligand 2. Am J Pathol.

[27] Pritzker KP, Gay S, Jimenez SA, Ostergaard K, Pelletier JP, Revell PA (2006). Osteoarthritis cartilage histopathology: grading and staging. Osteoarthr Cartil.

[28] Romero CA, Reed B, Mathew S, Brodi A, Dawood R, Twiner M (2018). Abstract P213: angiotensin converting enzyme inhibitors (ACEi) increase antifibrotic biomarkers in African American patients with hypertension and left ventricular hypertrophy. J Clin Hypertens (Greenwich).

[29] Rothkopf DM, Webb S, Szabo RM, Gelberman RH, May JW (1991). An experimental model for the study of canine flexor tendon adhesions. J Hand Surg Am.

[30] Rutherford RW, Jennings JM, Levy DL, Parisi TJ, Martin JR, Dennis DA (2018). Revision total knee arthroplasty for arthrofibrosis. J Arthroplast.

[31] Schindler R, Dinarello CA, Koch KM (1995). Angiotensin-converting-enzyme inhibitors suppress synthesis of tumour necrosis factor and interleukin 1 by human peripheral blood mononuclear cells. Cytokine.

[32] Tang Y, Hu X, Lu X (2015). Captopril, an angiotensin-converting enzyme inhibitor, possesses chondroprotective efficacy in a rat model of osteoarthritis through suppression local renin-angiotensin system. Int J Clin Exp Med.

[33] Uesugi T, Froh M, Gabele E, Isayama F, Bradford BU, Ikai I (2004). Contribution of angiotensin II to alcohol-induced pancreatic fibrosis in rats. J Pharmacol Exp Ther.

[34] Vignon E, Bejui J, Mathieu P, Hartmann JD, Ville G, Evreux JC (1987). Histological cartilage changes in a rabbit model of osteoarthritis. J Rheumatol.

[35] Wengrower D, Zanninelli G, Pappo O, Latella G, Sestieri M, Villanova A (2004). Prevention of fibrosis in experimental colitis by captopril: the role of tgf-beta1. Inflamm Bowel Dis.

[36] Xia Y, Sokhi UK, Bell RD, Pannellini T, Turajane K, Niu Y (2021). Immune and repair responses in joint tissues and lymph nodes after knee arthroplasty surgery in mice. J Bone Miner Res.

[37] Yan K, Shen Y (2017). Aliskiren has chondroprotective efficacy in a rat model of osteoarthritis through suppression of the local renin-angiotensin system. Mol Med Rep.

[38] Yoshiji H, Kuriyama S, Fukui H (2002). Angiotensin-I-converting enzyme inhibitors may be an alternative anti-angiogenic strategy in the treatment of liver fibrosis and hepatocellular carcinoma. Tumour Biol.

[39] Yoshioka M, Coutts RD, Amiel D, Hacker SA (1996). Characterization of a model of osteoarthritis in the rabbit knee. Osteoarthr Cartil.

[40] Zhang Y-Y, Yu Y, Yu C (2019). Antifibrotic roles of RAAS blockers: update. Renal fibrosis: mechanisms and therapies.

[41] Zhou H, Lu H (2021). Advances in the development of anti-adhesive biomaterials for tendon repair treatment. Tissue Eng Regen Med.

[42] Zhang YY, Yu Y, Yu C (2019) Antifibrotic Roles of RAAS Blockers: Update. In: Liu BC, Lan HY, Lv LL (eds) Renal Fibrosis: Mechanisms and Therapies. Advances in Experimental Medicine and Biology, vol 1165. Springer, Singapore. 10.1007/978-981-13-8871-2_33 10.1007/978-981-13-8871-2_33 PMC7121580 31399990

